# The Evidence Base for Circulating Tumor DNA-Methylation in Non-Small Cell Lung Cancer: A Systematic Review and Meta-Analysis

**DOI:** 10.3390/cancers16213641

**Published:** 2024-10-29

**Authors:** Debora Maffeo, Angela Rina, Viola Bianca Serio, Athina Markou, Tomasz Powrózek, Vera Constâncio, Sandra P. Nunes, Carmen Jerónimo, Alfonso Calvo, Francesca Mari, Elisa Frullanti, Diletta Rosati, Maria Palmieri

**Affiliations:** 1 Med Biotech Hub and Competence Center, Department of Medical Biotechnologies, University of Siena, 53100 Siena, Italy; debora.maffeo@dbm.unisi.it (D.M.); angela.rina@student.unisi.it (A.R.); viola.serio@dbm.unisi.it (V.B.S.); diletta.rosati2@unisi.it (D.R.); maria.palmieri@dbm.unisi.it (M.P.); 2 Cancer Genomics and Systems Biology Lab, Department of Medical Biotechnologies, University of Siena, 53100 Siena, Italy; 3 UOC Laboratorio di Assistenza e Ricerca Traslazionale, Dipartimento di Terapie cellulari, Ematologia e Medicina di Laboratorio, Azienda Ospedaliero-Universitaria Senese, 53100 Siena, Italy; francesca.mari@dbm.unisi.it; 4 Lab of Analytical Chemistry, Department of Chemistry, National and Kapodistrian University of Athens, 15772 Athens, Greece; atmarkou@chem.uoa.gr; 5 Department of Human Physiology, University of Lublin, 20080 Lublin, Poland; tomasz.powrozek@umlub.pl; 6 Cancer Biology and Epigenetics Group, Research Center (CI-IPOP)/CI-IPOP@RISE (Health Research Network), Portuguese Oncology Institute of Porto (IPO Porto)/Porto Comprehensive Cancer Center Raquel Seruca (Porto.CCC), 4200-072 Porto, Portugal; vera.salvado.constancio@ipoporto.min-saude.pt (V.C.); sandra.nunes@kuleuven.be (S.P.N.); carmenjeronimo@ipoporto.min-saude.pt (C.J.); 7 Department of Pathology and Molecular Immunology, ICBAS-School of Medicine & Biomedical Sciences, University of Porto, 4099-002 Porto, Portugal; 8 Program in Solid Tumors, CIMA, Cancer Center Clínica Universidad de Navarra (CCUN), Instituto de Investigación Sanitaria de Navarra (IDISNA), Department of Pathology, Anatomy and Physiology, School of Medicine, University of Navarra, 31008 Pamplona, Spain; acalvo@unav.es; 9 CIBERONC, ISCIII, 28029 Madrid, Spain

**Keywords:** circulating free DNA, methylation, non-small cell lung cancer, early diagnosis

## Abstract

*Background*: Non-Small Cell Lung Cancer (NSCLC) remains a challenging disease to manage with effectiveness. Early detection and precise monitoring are crucial for improving patient outcomes. Circulating tumor DNA (ctDNA) offers a non-invasive cancer detection and monitoring method. Emerging biomarkers, such as ctDNA methylation, have shown promise in enhancing diagnostic accuracy and prognostic assessment in NSCLC. In this review, we examined the current evidence regarding ctDNA methylation’s role in NSCLC detection through a systematic review of the existing literature and meta-analysis. *Methods*: We systematically searched PubMed, Medline, Embase, and Web of Science databases up to 26 June 2024 for studies on the role of ctDNA methylation analysis in NSCLC patients. We included studies from 2010 to 2024 on NSCLC patients. We excluded case reports, non-English articles, studies on cell lines or artificial samples, those without cfDNA detection, prognostic studies, and studies with non-extractable data or mixed cancer types. Funnel plots were visually examined for potential publication bias, with a *p* value < 0.05 indicating bias. Meta-analysis was conducted using R packages (meta, forestplot, and mada). Combined sensitivity, specificity, positive likelihood ratio (LR+), negative likelihood ratio (LR−), positive and negative predictive values, diagnostic odds ratio (DOR), and 95% confidence intervals (95% CI) were calculated. A summary receiver operating characteristic curve (SROC) and area under the curve (AUC) with related Standard Error (SE) were used to evaluate the overall diagnostic performance. Additionally, *RASSF1A*, *APC*, *SOX17*, *SEPT9,* and *RARβ2* were analyzed, since their methylation was assessed in two or more studies. *Results*: From 38 candidate papers, we finally identified 12 studies, including 472 NSCLC patients. The pooled sensitivity was 0.62 (0.47–0.77) and the specificity was 0.90 (0.85–0.94). The diagnostic odds ratio was 15.6 (95% CI 9.36–26.09) and the area under the curve was 0.249 (SE = 0.138). The positive and negative predictive values were 5.38 (95% CI 3.89–7.44) and 0.34 (95% CI 0.22–0.54), respectively. For single genes, the specificity reached 0.83~0.96, except for *RARβ2*, but the sensitivity was relatively low for each gene. Significant heterogeneity across the included studies, the potential publication bias for specificity (*p* = 0.0231), and the need to validate the clinical utility of ctDNA methylation for monitoring treatment response and predicting outcomes in NSCLC patients represent the main limitations of this study. *Conclusions:* These results provide evidence of the significant potential of ctDNA methylation as a valuable biomarker for improving the diagnosis of NSCLC, advocating for its integration into clinical practice to enhance patient management.

## Simple Summary

Non-Small Cell Lung Cancer (NSCLC) is the leading cause of cancer-related deaths and early detection is crucial for better outcomes. This study focuses on methylation, a new method for detecting and monitoring NSCLC using a blood test that looks for changes in DNA. These changes can be found in tiny fragments of tumor DNA circulating in the blood. By analyzing existing research, the authors found that this method is accurate and reliable, showing promise for early diagnosis and the better management of NSCLC patients. The findings suggest that this approach could be a valuable tool in clinical practice, potentially leading to improved survival rates by allowing for earlier and more precise treatment.

## 1. Introduction

Lung cancer is the leading cause of cancer-related mortality worldwide, with NSCLC accounting for approximately 85% of all cases [1,2,3]. NSCLC represents a significant challenge in clinical practice. Early diagnosis and precise monitoring are crucial for improving clinical outcomes in patients affected by this disease [4]. Traditional diagnostic methods, such as tissue biopsies, are invasive and often challenging due to tumor location and patient condition. Additionally, traditional biopsy carries inherent risks for the patient, including infection and complications, as well as diagnostic challenges such as insufficient material and sampling bias caused by tumor heterogeneity. In contrast to traditional biopsy, liquid biopsy is less invasive, poses fewer complications, allows for more consistent long-term monitoring, and enables rapid detection of clonal evolution and the emergence of resistance in cancer cells [5].

Liquid biopsies provide a non-invasive method for evaluating cancer by examining various circulating components. These include circulating nucleic acids such as circulating tumor DNA (ctDNA) and cell-free RNA (cfRNA), as well as circulating tumor cells (CTCs), microRNAs, extracellular vesicles, tumor-educated platelets, and methylated tumor-specific cell-free DNA [6]. In recent years, liquid biopsies have emerged as a promising alternative method for detecting and monitoring lung neoplasms, enabling real-time monitoring through a simple blood test [7,8] and overcoming the challenges arising from tumor heterogeneity.

In this context, ctDNA methylation has emerged as a potential biomarker for NSCLC, offering significant advantages over traditional diagnostic methods. DNA methylation in gene promoters is involved in the silencing of tumor suppressor genes in human lung cancers. This epigenetic modification, along with histone tail modifications, can alter chromatin condensation: protein complexes that bind methylated DNA and deacetylate histones lead to the formation of chromatin that represses transcription. In addition to genetic mutations, aberrant DNA methylation contributes to the inactivation of tumor suppressor genes, according to Knudson’s two-hit hypothesis. Several abnormally methylated genes have been identified in lung cancer, with methylation being an early event in tumorigenesis [9]. Studies indicate that DNA methylation changes can serve as diagnostic markers for specific cancer types or stages, underscoring the utility of DNA methylation as a molecular indicator. For instance, the methylation of the p16 promoter is proposed as a biomarker for the early detection of lung cancer and for monitoring prevention efforts [10,11].

Various methods are employed for ctDNA methylation analysis, including methylation-specific PCR (MSP), droplet digital PCR (ddPCR), and quantitative methylation-specific PCR (QMSP), each offering distinct levels of sensitivity and specificity. These differences can significantly impact the detection of low-abundance ctDNA, influencing the overall diagnostic accuracy. The choice of method is therefore a crucial factor in determining the reliability of ctDNA methylation as a biomarker, making it essential to consider these methodological variations when assessing diagnostic performance.

This systematic review aims to comprehensively examine the existing literature on ctDNA methylation in NSCLC, evaluating its clinical utility and potential impact on clinical practice. Current data regarding the diagnostic accuracy of ctDNA methylation will be analyzed to provide a comprehensive overview of the available evidence.

## 2. Materials and Methods

### 2.1. Search Strategy, Inclusion Criteria, and Data Collection

The meta-analysis was conducted adhering to PRISMA guidelines [12], systematically searching the PubMed, Medline, Embase, and Web of Science databases up to 26 June 2024 for studies reporting the role of ctDNA methylation analysis in NSCLC patients, using the combination of the following keywords: “circulating free dna” and “methylation” and “non small cell lung cancer”, without any restriction. Alternative spellings and abbreviations were considered during the review process. Eligible studies were evaluated by examining their titles and abstracts, while the references of all identified publications were checked to uncover any additional relevant studies that might have been missed initially. Manual searches were also conducted for pertinent reviews. Only those studies published in English and accessible in full text were included. The publications were reviewed for overlapping patient populations, and in instances where multiple articles from the same research group contained overlapping datasets, only the most significant or recent study was chosen for inclusion.

All the studies evaluating the application of ctDNA methylation analysis in NSCLC patients were considered eligible for the meta-analysis. The inclusion criteria were as follows: (i) studies published from 2010 to 2024; (ii) all NSCLC patients involved should be diagnosed cytologically or histopathologically; (iii) studies analyzing cancer detection/diagnosis/screening; (iv) methylation measured on cfDNA or ctDNA from blood plasma or serum.

During the evaluation of study eligibility, we initially eliminated case reports and articles published in languages other than English and prior to 2010. We then excluded studies that utilized cell lines or artificial samples, as well as those in which cfDNA was not detected. Additionally, studies that focused exclusively on the prognostic implications of cfDNA methylation were disregarded. Ultimately, studies that presented data in a manner that prevented proper extraction were also excluded from the final analysis. For instance, we omitted articles that contained mixed data from various cancer types beyond NSCLC or those concerning lung cancer without clear histological classification.

The information gathered from the eligible studies encompassed various characteristics, including the authors’ names, publication details (journal and date), study location, and patient numbers. Clinical data collected included the stage of cancer and smoking history, while results covered the cfDNA source and the methods used for methylation analysis, along with sensitivity and specificity metrics, with corresponding 95% confidence intervals. For studies providing only partial data, we obtained original information by reaching out to the corresponding authors. Patient-specific data were collected using a standardized form. Sensitivity was calculated as true positives divided by the sum of true positives and false negatives, while specificity was determined by dividing true negatives by the sum of true negatives and false positives. If such metrics were unavailable, we calculated the concordance rate as the ratio of the sum of true positives and true negatives to the total sample size, along with its 95% confidence interval.

To avoid bias, all records were reviewed by two authors independently (DM and AR), and a consensus was reached in each eligible study.

### 2.2. Methods for ctDNA Methylation Analysis

The analysis of ctDNA methylation employs various methods, each with distinct sensitivity and specificity profiles. The choice of methodology is critical for the accurate detection of methylation patterns and interpretation of clinical relevance. Methylation-specific PCR (MSP) and its variant, methylation-dependent MSP (mdMSP), are common techniques that use primers to differentiate between methylated and unmethylated sequences. While mdMSP improves specificity by targeting methylated cytosines, both methods are limited to predefined regions of interest.

Quantitative methylation-specific PCR (QMSP) enhances the sensitivity of conventional MSP by allowing the quantitative assessment of methylation levels in specific gene regions. Multiplex QMSP further improves efficiency by enabling the simultaneous quantification of multiple methylation targets. Droplet digital PCR (ddPCR) is highly sensitive, partitioning DNA into thousands of droplets, each undergoing PCR independently, which is advantageous for detecting low-abundance methylated ctDNA. Real-time PCR monitors the amplification of target DNA sequences in real-time, although it may struggle with low-input DNA samples or rare methylation variants. Amplification of Quantitative Analysis of Methylated Alleles-PCR (AQAMA-PCR) balances specificity and sensitivity by combining methylation specificity with real-time quantification.

Each method has its advantages and limitations regarding sensitivity, specificity, and the ability to detect low levels of ctDNA in plasma samples. Therefore, the selection depends on the study’s aims, the quantity of ctDNA, and the regions of interest targeted for methylation analysis.

### 2.3. Statistical Methods

Meta-analysis was carried out using the R packages (version 4.4.1) *meta* (version 7.0), *forestplot* (version 3.1.3), and *mada* (version 0.5.11). For each eligible study, we calculated various metrics, including the combined sensitivity, specificity, positive likelihood ratio (LR+ = sensitivity divided by (1-specificity)), negative likelihood ratio (LR− = (1-sensitivity) divided by specificity), positive predictive value, negative predictive value, and the diagnostic odds ratio (DOR = LR+/LR−). Additionally, we computed the corresponding 95% confidence intervals (95% CI) for these values.

A bivariate random-effects model using the Reitsma approach was applied to assess the diagnostic accuracy of the cfDNA test across multiple studies. This model considers the correlation between sensitivity and specificity, while accounting for heterogeneity between studies. A summary receiver operating characteristic (SROC) curve was generated, and the area under the curve (AUC) was computed to evaluate overall diagnostic performance. Q*, representing the point where sensitivity equals specificity, was used to measure the balance between detecting true positives and minimizing false positives. Funnel plots were visually examined for potential publication bias, with a *p* value < 0.05 indicating the presence of such bias.

## 3. Results

### 3.1. Study Selection

**Figure 1:**
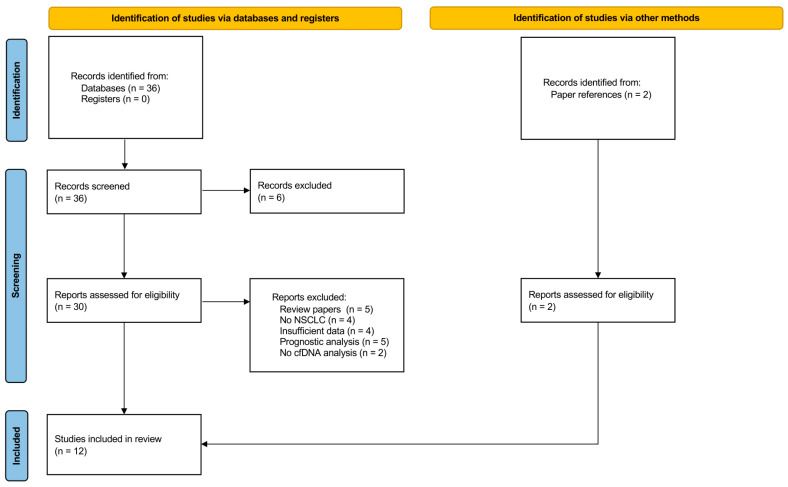
PRISMA 2020 flow diagram of literature screening and study selection.

Initially, 38 studies were identified as potential candidates for the meta-analysis based on the bibliographic search results. Following a preliminary screening of titles and abstracts, 32 full-text articles were chosen for a more detailed assessment of their eligibility and were thoroughly reviewed. The primary reasons for exclusion included the articles being reviews, not focusing on NSCLC, lacking an evaluation of cfDNA, providing insufficient data, or solely analyzing prognostic factors. After excluding studies, a total of 12 eligible studies were identified and included in our meta-analysis. A flowchart of the literature selection is shown in Figure 1.

### 3.2. Characteristics of Eligible Studies

The twelve eligible studies for meta-analysis were published between 2010 and 2024 and included 472 NSCLC patients from eight countries. In the case of multiple publications from the same research group of overlapping patient populations, only the largest cohort was selected [13,14,15,16,17,18,19].

The mean number of patients for each study was 59 (range 38–110). Various methods were applied to detect cfDNA methylation, and the quantitative methylation-specific polymerase chain reaction (QMSP) was the most common method (7/12). The median age was 63.5 years (range 30–89), 69.3% of patients were male, and 79.4% had a history of smoking (former or current). Most of the patients were at an advanced stage (TNM III–IV). All publications were focused on ctDNA in plasma. The main characteristics of the 12 included studies are shown in Table 1.

### 3.3. Diagnostic Accuracy

**Figure 2:**
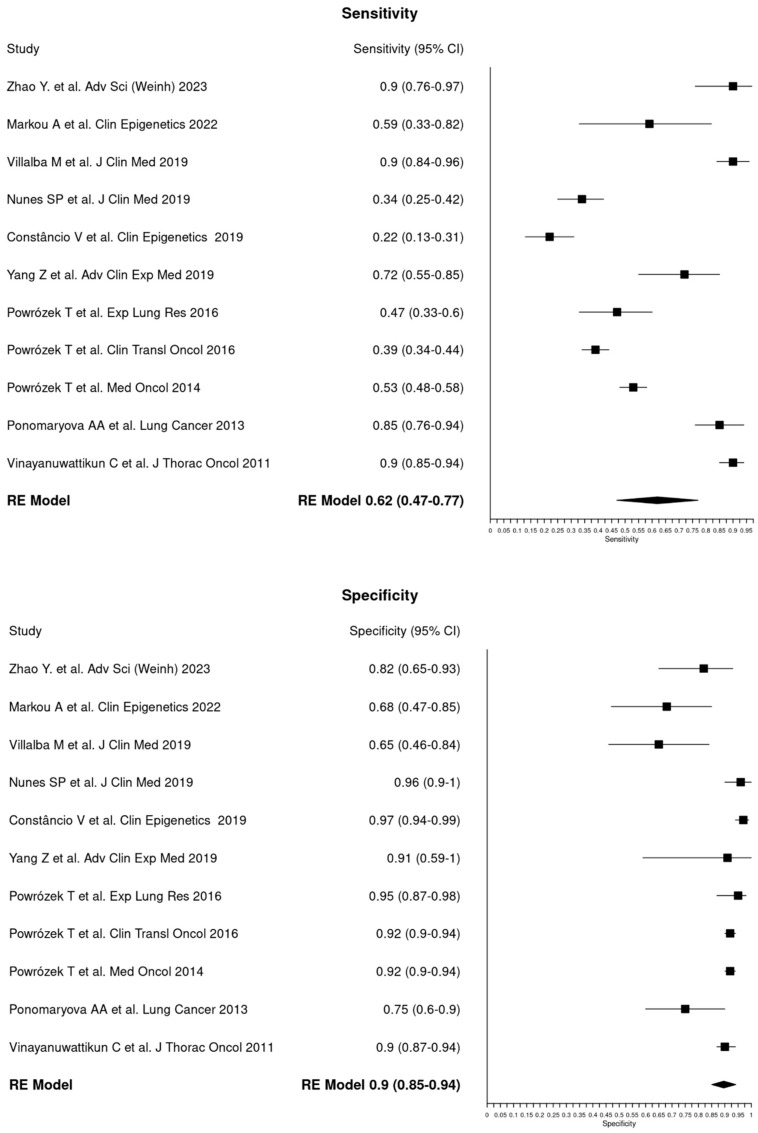
**A** paired forest plot illustrating the sensitivity and specificity of cfDNA methylation in NSCLC across 11 studies included in the meta–analysis [13,14,15,16,17,18,20,21,22,23,24]. A random–effects (RE) model was employed for the analysis. Each square and horizontal bar denotes the sensitivity and specificity for individual studies, accompanied by a 95% confidence interval (CI). The diamonds represent the overall findings, with the pooled sensitivity calculated as 0.62 (ranging from 0.47 to 0.77) and the pooled specificity at 0.90 (ranging from 0.85 to 0.94).

Out of 32 assessed eligible papers, five were excluded because of review papers [6,25,26,27,28], four because no NSCLC [29,30,31,32], four were excluded since no sufficient data were reported in the published data and the raw data were not possible to obtain [33,34,35,36], five were because of prognostic papers [37,38,39,40,41], and two were because they did not concern cfDNA [42,43]. For the calculation of diagnostic accuracy, the number of patients and controls used in each study to determine sensitivity values, specificity, and corresponding 95% CIs for the genes assessed for cfDNA methylation was considered. Of the 12 remaining studies, one [18] was excluded because the same group had already analyzed the same gene in a more recent study involving overlapping populations [19]. Thus, the diagnostic accuracy of cfDNA methylation in NSCLC shown in the Forest Plot (Figure 2) was ultimately calculated from 11 studies, including 651 NSCLC patients and 604 controls. The majority of publications examined the methylation of cfDNA of a combination of genes (*n* = 7), though the remaining studies examined the methylation of single genes (*n* = 4). Among the latter, genes commonly analyzed for methylation were *RASSF1A*, *APC*, *SOX17*, *SEPT9,* and *RARβ2.* The combined sensitivity and specificity of overall methylation in the meta-analysis were 0.62 (0.47–0.77) and 0.90 (0.85–0.94) (Table 2).

The volume of plasma used for cfDNA extraction varied among the studies included in this review. Some studies reported extracting cfDNA from as little as 0.5 mL of plasma, while others used 1 mL, 2 mL, or larger volumes, such as between 0.5 and 2 mL, or between 2 and 3 mL. A few studies even utilized a minimum plasma volume of 3.5 mL or 4 mL.

Sensitivity results showed similar variation. In studies using 0.5 mL of plasma, the sensitivity was reported as 0.72. For studies extracting cfDNA from 1 mL of plasma, sensitivity values were 0.90, 0.90, and 0.85. When 2 mL of plasma was used, the sensitivity ranged from 0.34 to 0.59. Studies that extracted cfDNA from 0.5 to 2 mL of plasma reported a sensitivity of 0.90, while those using between 2 and 3 mL showed a sensitivity of 0.22. In studies with a minimum plasma volume of 3.5 mL and 4 mL, the sensitivities were 0.39 and 0.53, respectively.

This variability in plasma volume and sensitivity underscores the need to carefully consider these factors in the analysis of cfDNA methylation studies.

**Figure 3:**
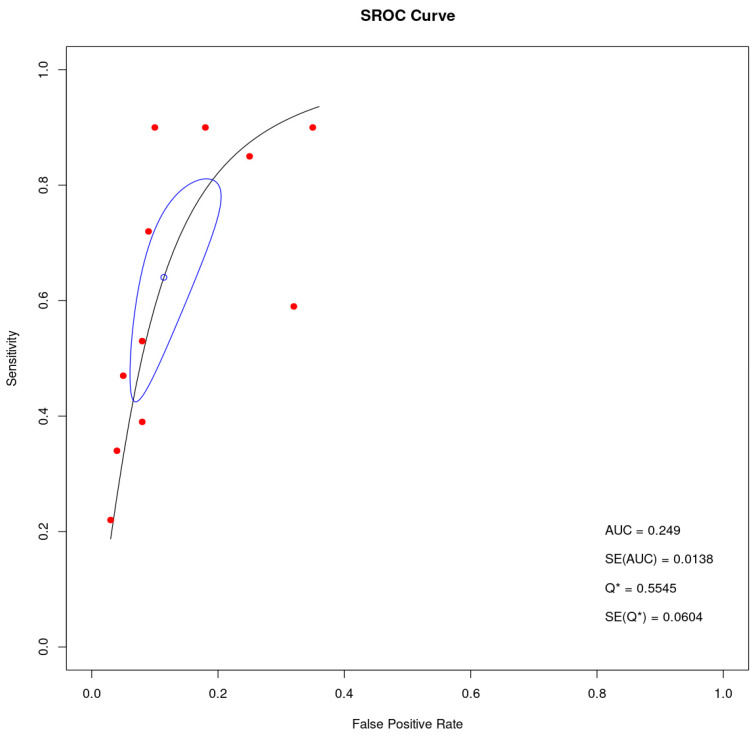
Summary receiver operating characteristic (SROC) plot. The curve illustrates the diagnostic accuracy of the cfDNA test across multiple studies. The red points represent individual study results, while the black curve summarizes the overall diagnostic performance. Q* is the point on the curve where sensitivity equals specificity, providing a measure of the test’s balance between detecting true positives and minimizing false positives. The SE(Q*) is the standard error of Q*.

The LR+ and LR− of cfDNA were 5.38 (95% CI 3.89–7.44) and 0.34 (95% CI 0.22–0.54), respectively, in the meta-analysis. The DOR was 15.6 (95% CI 9.36–26.09). Figure 3 showed the summary receiver operating characteristic (SROC) plot with an AUC of 0.249 (SE = 0.0138), indicating a limited diagnostic accuracy of cfDNA test. However, this result reflects that the cfDNA test’s accuracy is limited by the variability in the studies, which affects the overall diagnostic performance of the test.

We then performed the same analysis focusing on individual genes. We considered genes analyzed for methylation in at least two independent studies, namely *RASSF1A*, *APC*, *SOX17*, *SEPT9,* and *RARβ2* (Table 3).

**Figure 4:**
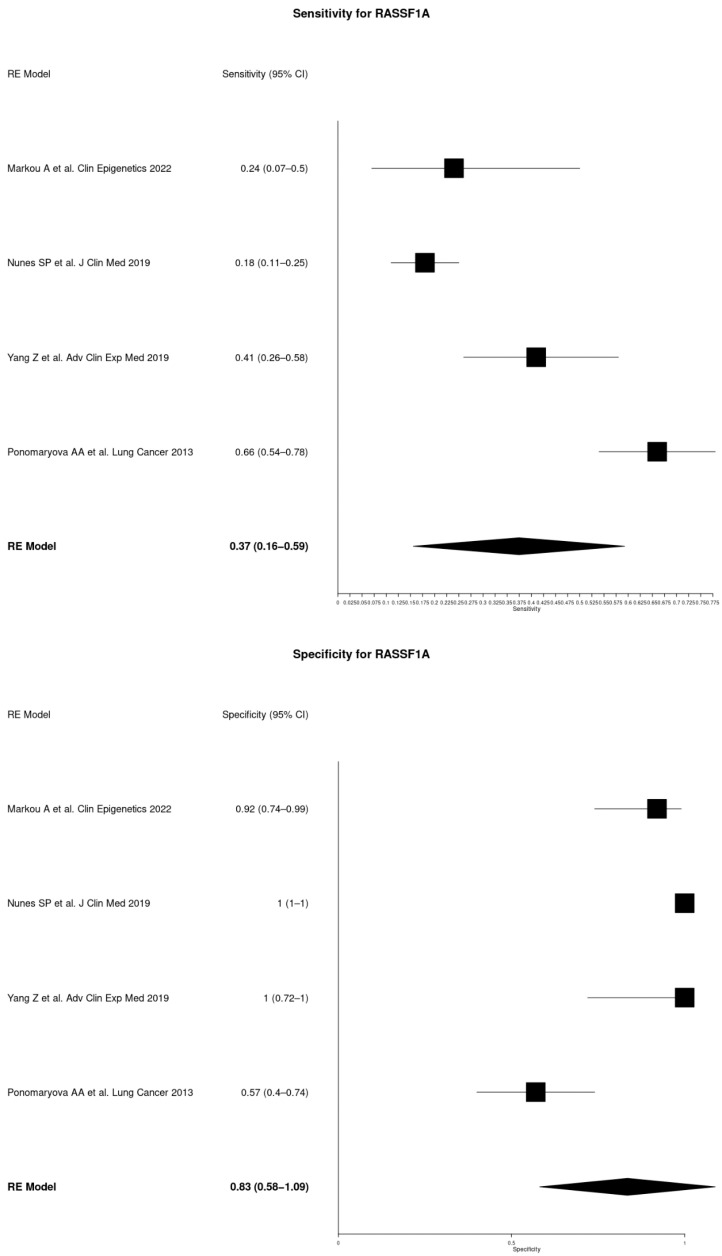

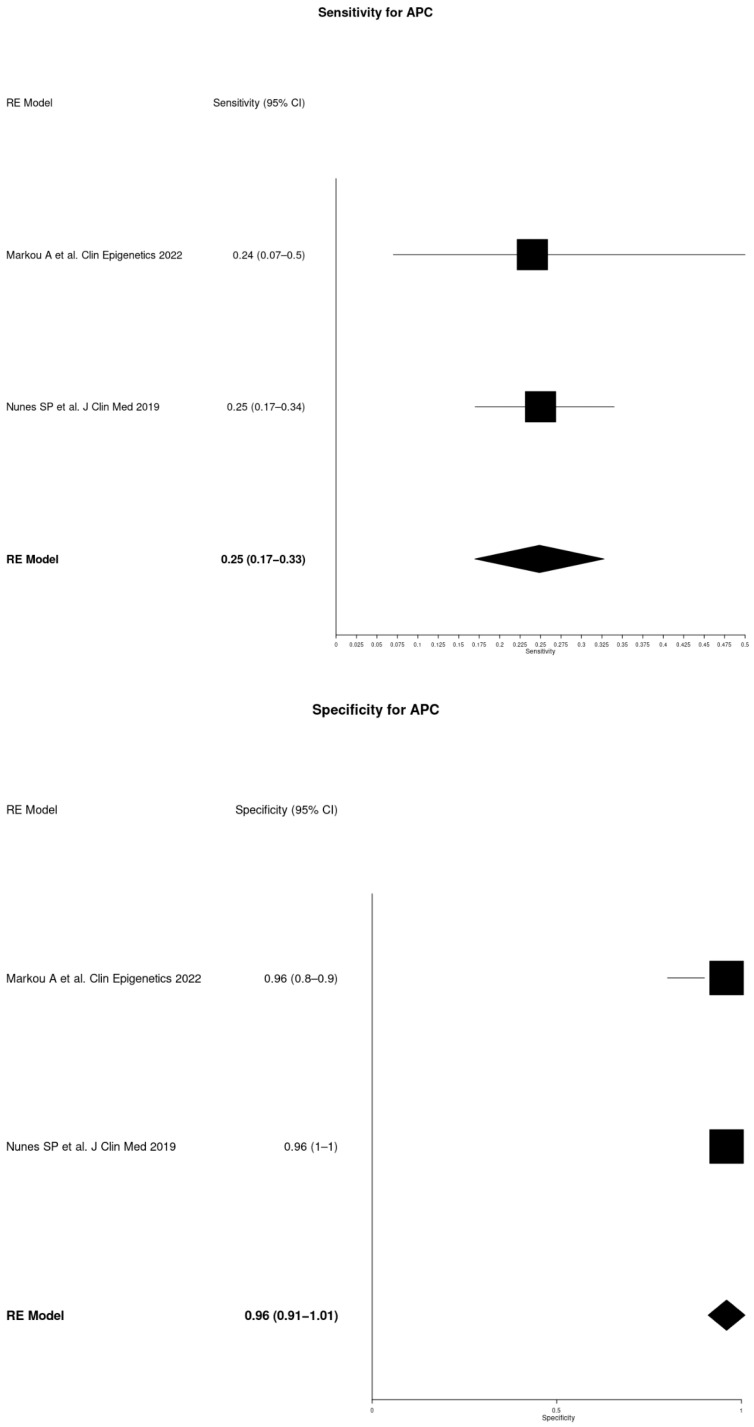

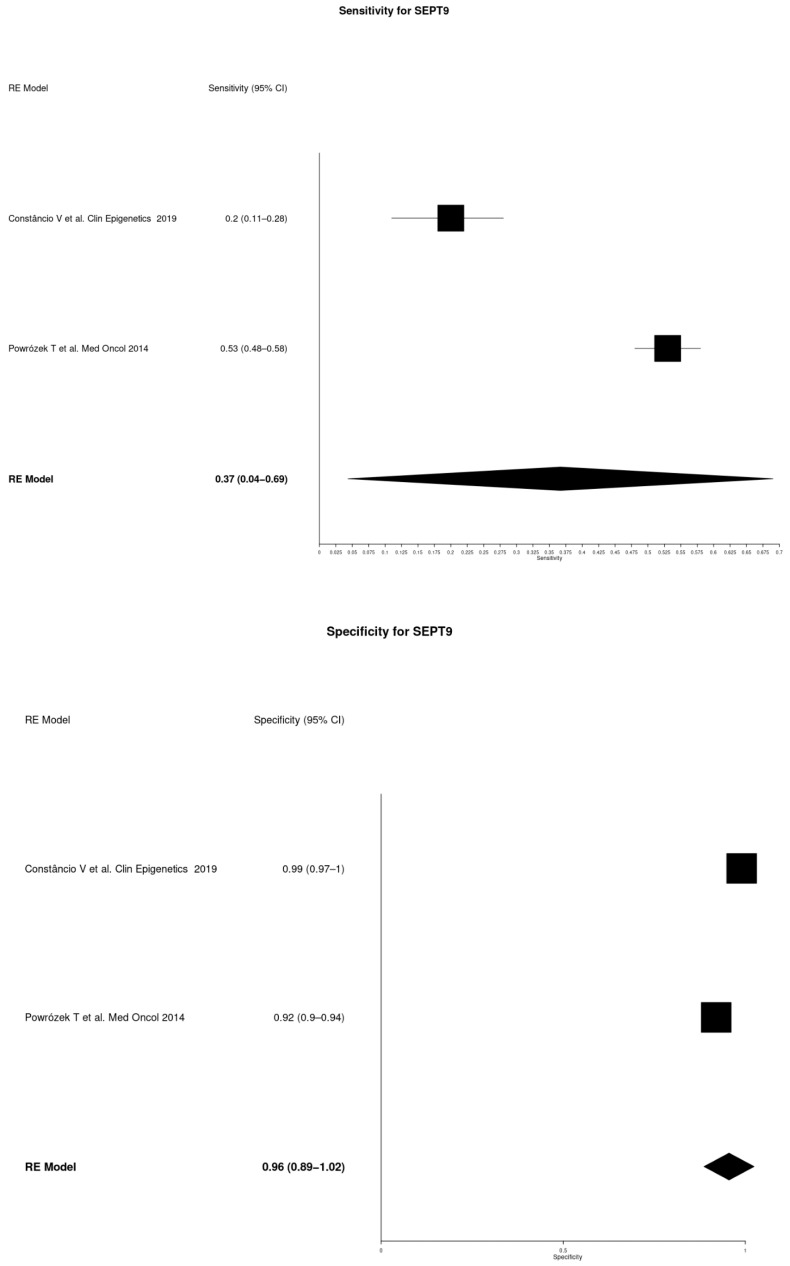

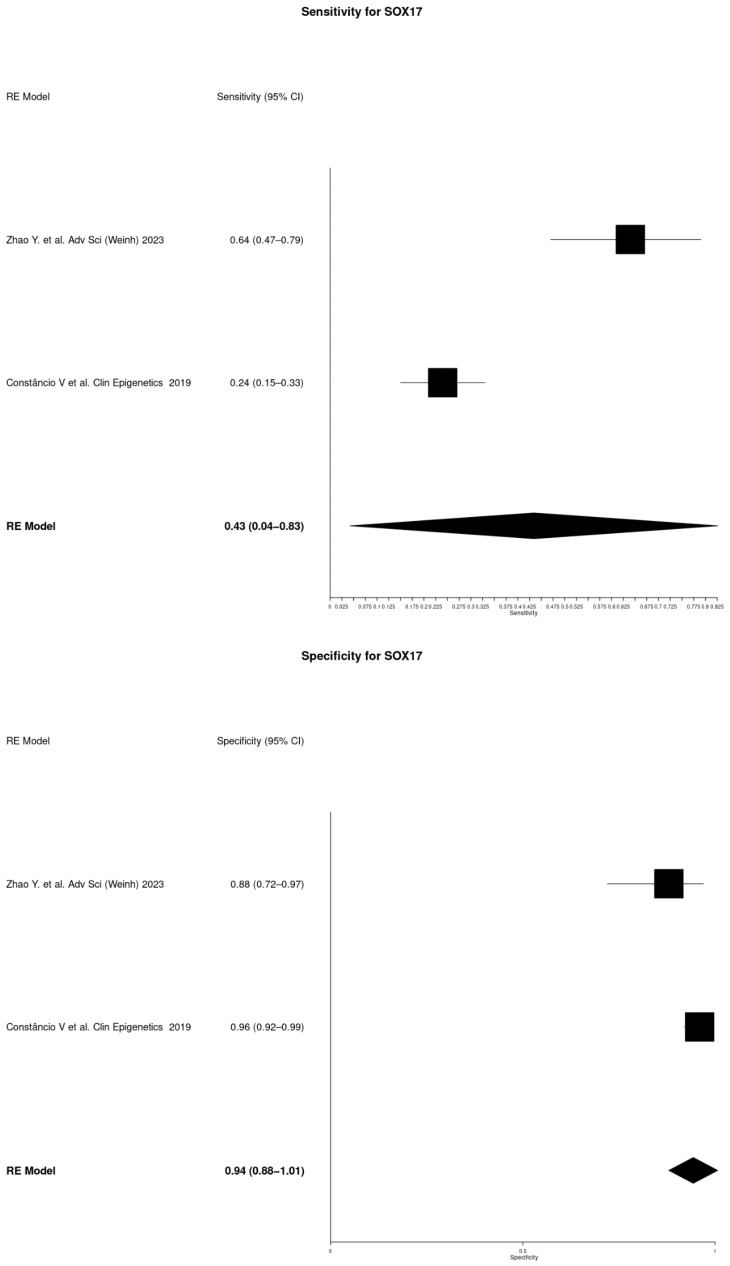

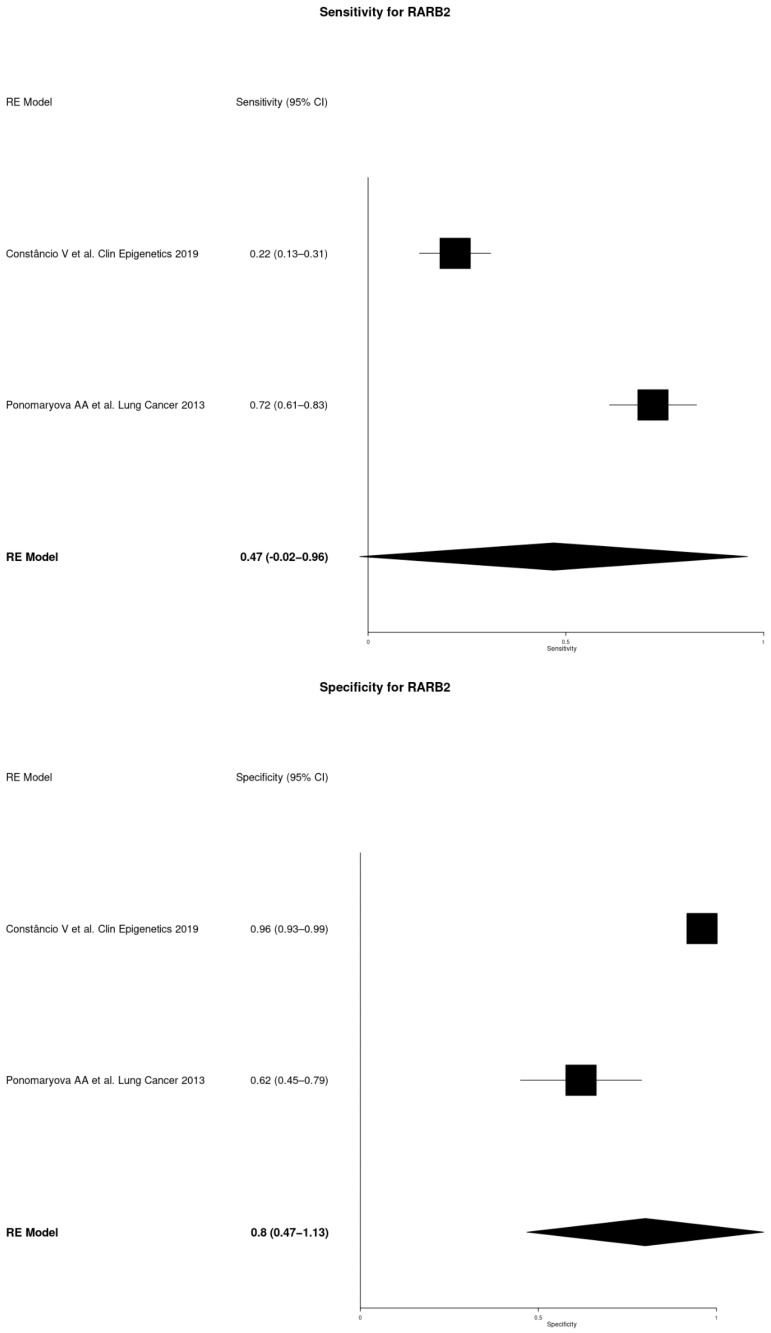
Paired Forest Plot of sensitivity and specificity of genes assessed for methylation in two or more studies included in meta-analysis: *RASSF1A*, *APC*, *SOX17*, *SEPT9,* and *RARβ2* [13,14,17,18,20,21,23].

The sensitivity and specificity for *RASSF1A*, *APC*, *SOX17,* and *SEPT9* were 0.37 (95% CI 0.16–0.59) and 0.83 (95% CI 0.58–1.09), 0.25 (95% CI 0.17–0.33) and 0.96 (95% CI 0.91–1.01), 0.43 (95% CI 0.04–0.83) and 0.94 (95% CI 0.88–1.01), and 0.37 (95% CI 0.04–0.69) and 0.94 (95% CI 0.89–1.02), respectively (Figure 4; Table 4). For *RARβ2*, no significance was identified for both sensitivity and specificity (0.47 (95% CI −0.02–0.96) and 0.8 (95% CI 0.47–1.13).

### 3.4. Heterogeneity and Publication Bias

**Figure 5:**
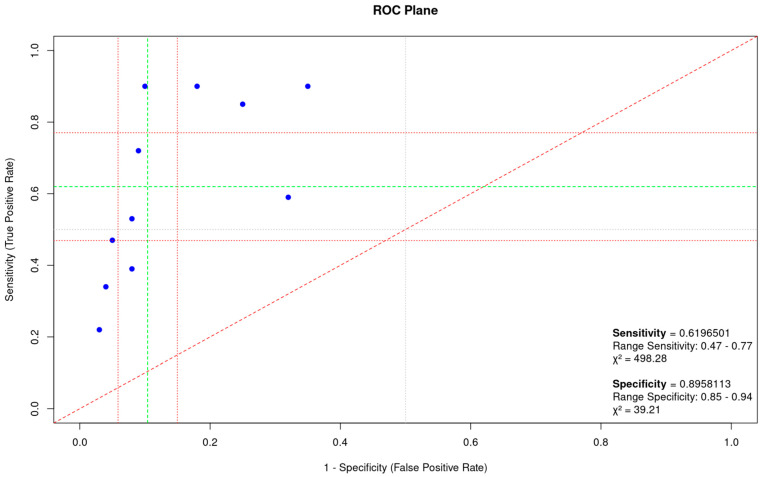
ROC plane of sensitivity and specificity of cfDNA methylation in NSCLC for 11 studies included in meta–analysis [13,14,15,16,17,18,20,21,22,23,24].

The threshold effect is a significant contributor to heterogeneity among studies. An examination of the ROC plane indicated no notable threshold effect (Figure 5).

**Figure 6:**
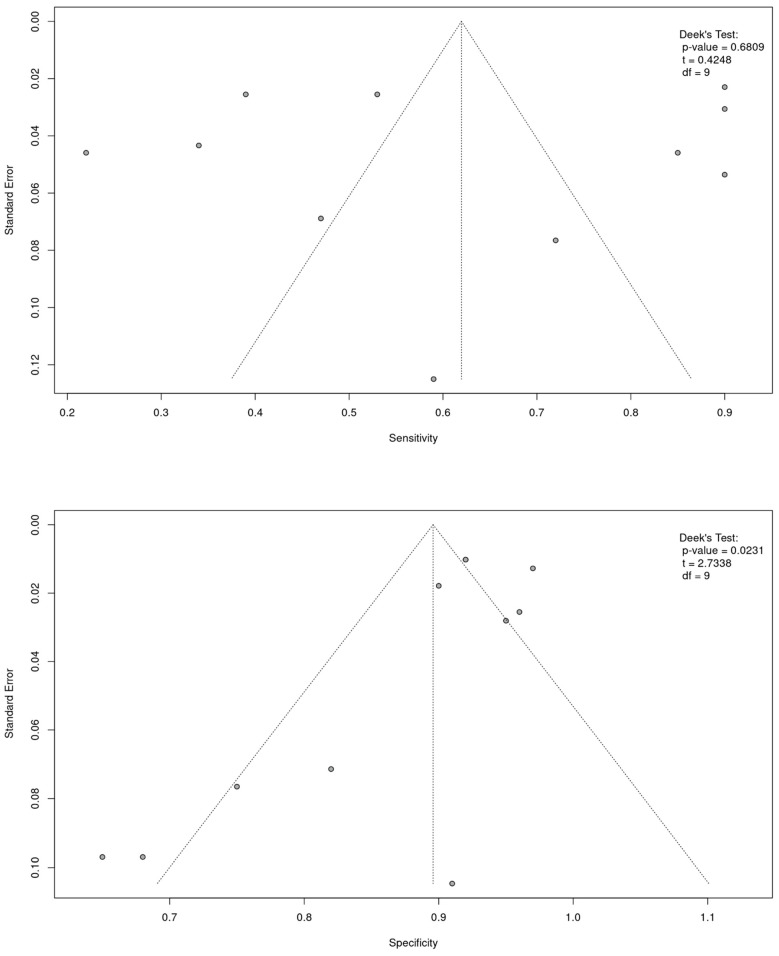
**A** funnel plot of the sensitivity and specificity of cfDNA methylation in NSCLC for the 11 studies included in the meta-analysis [13,14,15,16,17,18,20,21,22,23,24]. Deek’s test showed a *p*-value of 0.0231 for specificity and a *p*-value of 0.6809 for sensitivity.

To assess publication bias, we utilized a funnel plot (Figure 6). Visual analysis suggested partial symmetry, with *p*-values of 0.6809 for sensitivity and 0.0231 for specificity. This indicates no evidence of publication bias for sensitivity, while a weak publication bias was observed for specificity.

The ROC plane plots the sensitivity against specificity (false positive rate), allowing the visualization of the diagnostic performance across studies. The red diagonal line represents the line of no-discrimination. The average sensitivity was 0.6197 (range: 0.47–0.77) and the average specificity was 0.8958 (range: 0.85–0.94). The chi-square (χ^2^) indicates the measure of discrepancy between observed and expected data, with high values suggesting the presence of significant heterogeneity among the studies analyzed.

## 4. Discussion

This systematic review and meta-analysis assessed the diagnostic performance of circulating tumor DNA (ctDNA) methylation as a biomarker for Non-Small Cell Lung Cancer (NSCLC). The results indicate that ctDNA methylation has considerable promise as a non-invasive method for the early detection and monitoring of NSCLC, demonstrating pooled sensitivity and specificity values of 0.62 (ranging from 0.47 to 0.77) and 0.90 (ranging from 0.85 to 0.94), respectively. The diagnostic odds ratio (DOR) of 15.6 (95% CI 9.36–26.09) and the area under the curve (AUC) of 0.249 (SE = 0.0138) highlight a discrepancy between the test’s performance at an optimal threshold and its overall ability to correctly identify NSCLC cases across multiple thresholds. The high DOR suggests that ctDNA methylation is effective in distinguishing NSCLC patients from controls when a specific cut-off is used. However, the low AUC reveals that the test performs inconsistently across a broader range of thresholds. As a result, while ctDNA methylation shows promise in certain controlled settings, its overall reliability as a diagnostic tool for NSCLC is limited when applied more broadly. The combined sensitivity of 61.97% indicates that the evaluation of ctDNA methylation can correctly identify approximately two-thirds of disease cases (true positives), though with moderate accuracy. The combined specificity of 89.58% suggests that ctDNA methylation is highly effective in identifying healthy individuals (true negatives), demonstrating a strong ability to avoid false positives. However, the large χ^2^ value for sensitivity (498.28) indicates substantial heterogeneity among the studies, particularly for sensitivity, which could affect the overall reliability of these estimates.

Tumor heterogeneity, especially in the metastatic phase, is influenced by various factors including histological subtypes (such as adenocarcinoma and squamous cell lung cancer) and genetic mutations (such as those in *EGFR*, *BRAF*, *KRAS*, and *ALK* translocations). These variations contribute to significant interpersonal and intratumoral diversity, depending on the histotype and tumor grade. However, most of the studies analyzed did not provide detailed information on these genetic and histological characteristics, nor did they consider their impact on cfDNA methylation results. This lack of detailed information may partially explain the discrepancies observed in our findings compared to data reported by other authors.

The sensitivity of cfDNA methylation analysis seems to be influenced by the volume of plasma used, with higher sensitivities generally found in studies using smaller volumes, though this trend is not consistent across all studies. Other factors, such as the specific genes analyzed and the detection methods employed, also impact sensitivity. Given the variability in methods and plasma volumes across studies, standardization in future research is needed to better understand how plasma volume affects the sensitivity of ctDNA methylation analysis.

These findings align with previous studies that have highlighted the value of ctDNA methylation as a biomarker for lung cancer detection. Unlike traditional tissue biopsies, which can be invasive and limited by tumor heterogeneity, ctDNA methylation offers a more comprehensive assessment of the tumor genome from a simple blood sample. This makes it especially valuable in clinical settings where repeated sampling is required, or where tissue biopsies are not feasible due to tumor location or patient condition.

The high specificity observed suggests that ctDNA methylation is particularly effective in correctly identifying patients without the disease, minimizing false positives. This is crucial in screening high-risk populations, such as heavy smokers, to avoid unnecessary invasive procedures or psychological distress. However, the sensitivity of 62% indicates that a significant proportion of NSCLC cases might still go undetected using ctDNA methylation alone. Therefore, combining ctDNA methylation with other diagnostic tools, such as imaging or molecular biomarkers, could enhance overall detection rates.

The analysis also revealed variability in diagnostic performance across different methylation markers. Table 4 presents combined sensitivity and specificity values for several genes assessed for methylation in at least two independent studies. *RASSF1A* has a sensitivity of 0.37 (95% CI: 0.16–0.59) and a specificity of 0.83 (95% CI: 0.58–1.09). *APC* shows a sensitivity of 0.25 (95% CI: 0.17–0.33) and a specificity of 0.96 (95% CI: 0.91–1.01). *SOX17* has a sensitivity of 0.43 (95% CI: 0.04–0.83) and a specificity of 0.94 (95% CI: 0.88–1.01). *SEPT9* demonstrates a sensitivity of 0.37 (95% CI: 0.04–0.69) and a specificity of 0.96 (95% CI: 0.89–1.02). For *RARB2*, no significance was identified for both sensitivity and specificity (0.47 (95% CI −0.02–0.96) and 0.8 (95% CI 0.47–1.13).

Among these markers, *APC* appears to be the most specific (0.96), with a relatively narrow confidence interval, indicating high reliability in correctly identifying non-diseased individuals. However, its sensitivity is relatively low (0.25), meaning it may miss a significant number of true positive cases. *SOX17* presents the highest sensitivity (0.43), suggesting it has the potential to identify more true positive cases compared to the other genes, though its specificity is slightly lower (0.94). Despite these individual performances, relying on a single biomarker is not ideal due to the limitations in sensitivity and specificity. A panel of genes, combining the strengths of multiple markers, would likely offer a more comprehensive and reliable diagnostic tool. Utilizing a combination of these methylation markers, possibly alongside other biomarkers or diagnostic tests, can enhance overall accuracy, providing a better balance between sensitivity and specificity.

The choice of methods for ctDNA methylation analysis significantly impacts the sensitivity and specificity of the results. Techniques like ddPCR offer high sensitivity for detecting low-abundance ctDNA, but they can be expensive and technically challenging to implement on a large scale. Conversely, methods such as QMSP and multiplex QMSP allow for the quantification of multiple regions of interest, though they may be less effective when ctDNA levels are low.

Technical limitations, such as low sensitivity, can lead to false negatives, especially in early-stage disease or when ctDNA is scarce. On the other hand, more specific methods like AQAMA-PCR and mdMSP reduce the risk of false positives but are restricted to predefined genomic regions.

It is crucial to consider how the variability in these methods affects the comparability of studies, underscoring the need for standardization to ensure reproducible and reliable results. Future research should focus on optimizing and integrating these approaches to enhance their clinical applicability.

This approach can lead to more effective detection and diagnosis, reducing the chances of false positives and false negatives.

One of the key strengths of this meta-analysis is the large number of patients included, which enhances the reliability of the pooled estimates. However, several limitations need to be addressed. First, significant heterogeneity was observed across the included studies. Although meta-regression analyses did not identify specific covariates (such as country, study design, sample size, or detection methods) as contributors to this heterogeneity, likely differences in patient populations, sample processing techniques, and ctDNA methylation detection methods played a role. Future studies should aim for standardization in methodology to reduce heterogeneity and improve the comparability of results across studies.

Another limitation is the potential for publication bias for specificity (*p* value = 0.0231). This could be because studies with negative results were underreported or not published, which could skew the meta-analysis findings toward more favorable results.

In terms of clinical application, while ctDNA methylation holds promise, there are still challenges to be addressed before it can be widely implemented in clinical practice. The cost of ctDNA assays, the need for highly sensitive detection methods, and the interpretation of results in the context of tumor heterogeneity and clonal evolution are important considerations. Moreover, prospective clinical trials are necessary to validate the clinical utility of ctDNA methylation for monitoring treatment response and predicting outcomes in NSCLC patients.

## 5. Conclusions

In conclusion, this systematic review and meta-analysis provide strong evidence supporting the use of ctDNA methylation as a diagnostic biomarker in NSCLC. Its high specificity, coupled with moderate sensitivity, makes it a valuable tool for early detection, particularly in high-risk populations. However, further research is needed to refine its application, standardize detection methods, and explore its prognostic potential. Integrating ctDNA methylation analysis into clinical practice has the potential to revolutionize NSCLC management, offering a less invasive, yet highly informative, approach to cancer detection and monitoring.

## Figures and Tables

**Table 1:** Characteristics of eligible studies included in meta- and pooled analyses.

Reference	No. of NSCLC Cases	Country	Males/Females	Median Age (Range)	% Smoker (Former or Current)	NSCLC Stage (I–IV)	Specimen Type for ctDNA	Detection Assay in ctDNA
Zhao Y. et al. *Adv. Sci. (Weinh)* 2023 [20]	39	United States	18/21	68 (30–85)	97.4	I, II, III, IV	Plasma	mdMSP
Markou A et al. *Clin Epigenetics* 2022 [21]	42	Greece	32/10	69 (39–89)	78.6	IA–IIIA	Plasma	QMSP
Villalba M et al. *J Clin Med* 2019 [22]	89	Spain	66/23	61.5 (30–86)	83.1	I, II, III, IV	Plasma	ddPCR
Nunes SP et al. *J Clin Med* 2019, Constâncio V et al. *Clin Epigenetics* 2019 [13,14]	110	Portugal	75/35	66.5 (38–89)	75.5	I, II, III, IV	Plasma	QMSP, multiplex QMSP
Yang Z et al. *Adv Clin Exp Med* 2019 [23]	39	China	24/15	51 (NA)	74.4	I	Plasma	QMSP
Powrózek T et al. *Exp Lung Res* 2016, Powrózek T et al. *Clin Transl Oncol* 2016, Powrózek T et al. *Med Oncol* 2014 [15,16,17]	55	Poland	40/15	62 (NA)	88.6	I, II, IIIA, IIIB, IV	Plasma	QMSP, real-time PCR
Ponomaryova AA et al. *Lung Cancer* 2013, Ponomaryova AA et al. *Eur J Cancer Prev* 2011 [18,19]	60	Russia	52/8	NA	83.0	I, II, III	Plasma	QMSP
Vinayanuwattikun C et al. *J. Thorac. Oncol.* 2011 [24]	38	Thailand	20/18	NA	50.0	III, IV	Plasma	AQAMA-PCR
Total of cases	472							

NA, not available; MCED, multi-cancer early detection; MRD, minimal residual disease; timMRD, tumor-informed methylation-based MRD; PCR, polymerase chain reaction; ddPCR, droplet digital PCR; MSP, methylation-specific PCR; mdMSP, multiplex digital MSP; QMSP, quantitative MSP; 5hmC, 5-Hydroxymethylcytosine; AQAMA, absolute quantitative analysis of methylated alleles.

**Table 2:** Synthesis of studies included in meta-analysis for genes assessed for cfDNA methylation.

Reference	Gene	N° NSCLC Patients	N° Controls	Sensitivity	95% CI Sensitivity	Specificity	95% CI Specificity
Zhao Y. et al. *Adv. Sci. (Weinh)* 2023 [20]	*SOX17*, *CDO1*, *TAC1*, *HOXA7*	39	33	0.90	0.76–0.97	0.82	0.65–0.93
Markou A et al. *Clin Epigenetics* 2022 [21]	*APC*, *RASSF1A*, *FOXA1*, *SLFN11*, *SHOX2*	42	12	0.59	0.33–0.82	0.68	0.47–0.85
Villalba M et al. *J Clin Med* 2019 [22]	*TMPRSS4*	89	25	0.90	0.84–0.96	0.65	0.46–0.84
Nunes SP et al. *J Clin Med* 2019 [13]	*APC*, *RASSF1A*	110	28	0.34	0.25–0.42	0.96	0.9–1
Constâncio V et al. *Clin Epigenetics* 2019 [14]	*RARβ2*, *SEPT9*, *SOX17*	86	136	0.22	0.13–0.31	0.97	0.94–0.99
Yang Z et al. *Adv Clin Exp Med* 2019 [23]	*CDH13*, *WT1*, *CDKN2A*, *HOXA9*, *PITX2*, *CALCA*, *RASSF1A*, *DLEC1*	39	11	0.72	0.55–0.85	0.91	0.59–1
Powrózek T et al. *Exp Lung Res* 2016 [15]	*RTEL1*, *PCDHGB6*	55	80	0.47	0.33–0.60	0.95	0.87–0.98
Powrózek T et al. *Clin Transl Oncol* 2016 [16]	*DCLK1*	46	95	0.39	0.34–0.44	0.92	0.9–0.94
Powrózek T et al. *Med Oncol* 2014 [17]	*SEPT9*	47	100	0.53	0.48–0.58	0.92	0.90–0.94
Ponomaryova AA et al. *Lung Cancer* 2013 [18]	*RARβ2*, *RASSF1A*	60	32	0.85	0.76–0.94	0.75	0.6–0.9
Vinayanuwattikun C et al. *J Thorac Oncol* 2011 [24]	*SHP1P2*	38	52	0.90	0.85–0.94	0.90	0.87–0.94
Total cases		651	604	0.62	0.47–0.77	0.90	0.85–0.94

**Table 3:** A summary of the genes assessed for methylation in at least two independent studies included in the meta-analysis.

Gene	Reference	N° NSCLC Patients	N° Controls	Sensitivity	95% CI Sensitivity	Specificity	95% CI Specificity
*RASSF1A*	Markou A et al. *Clin Epigenetics* 2022 [21]	42	12	0.24	0.07–0.50	0.92	0.74–0.99
	Nunes SP et al. *J Clin Med* 2019 [13]	110	28	0.18	0.11–0.25	1	1–1
	Yang Z et al. *Adv Clin Exp Med* 2019 [23]	39	11	0.41	0.26–0.58	1	0.72–1
	Ponomaryova AA et al. *Lung Cancer* 2013 [18]	60	32	0.66	0.54–0.78	0.57	0.40–0.74
*APC*	Markou A et al. *Clin Epigenetics* 2022 [21]	42	12	0.24	0.07–0.50	0.96	0.80–1
	Nunes SP et al. *J Clin Med* 2019 [13]	110	28	0.25	0.17–0.34	0.96	0.90–1
*SOX17*	Zhao Y. et al. *Adv Sci (Weinh)* 2023 [20]	39	33	0.64	0.47–0.79	0.88	0.72–0.97
	Constâncio V et al. *Clin Epigenetics* 2019 [14]	86	136	0.24	0.15–0.33	0.96	0.92–0.99
*SEPT9*	Constâncio V et al. *Clin Epigenetics* 2019 [14]	86	136	0.20	0.11–0.28	0.99	0.97–1
	Powrózek T et al. *Med Oncol* 2014 [17]	47	100	0.53	0.48–0.58	0.92	0.90–0.94
*RARβ2*	Constâncio V et al. *Clin Epigenetics* 2019 [14]	86	136	0.22	0.13–0.31	0.96	0.93–0.99
	Ponomaryova AA et al. *Lung Cancer* 2013 [18]	60	32	0.72	0.61–0.83	0.62	0.45–0.79

**Table 4:** Combined sensitivity and specificity values for genes assessed for methylation in two or more studies.

Gene	Sensitivity	95% CI Sensitivity	Specificity	95% CI Specificity
*RASSF1A*	0.37	0.16–0.59	0.83	0.58–1.09
*APC*	0.25	0.17–0.33	0.96	0.91–1.01
*SOX17*	0.43	0.04–0.83	0.94	0.88–1.01
*SEPT9*	0.37	0.04–0.69	0.96	0.89–1.02
*RARβ2*	0.47	−0.02–0.96	0.80	0.47–1.13

## Data Availability

The original contributions presented in the study are included in the article; further inquiries can be directed to the corresponding author/s.
